# Prognostic and clinicopathological role of long non-coding RNA UCA1 in various carcinomas

**DOI:** 10.18632/oncotarget.16059

**Published:** 2017-03-09

**Authors:** Xiaoxiong Wang, Fei Peng, Liang Cheng, Guang Yang, Daming Zhang, Jiaqi Liu, Xin Chen, Shiguang Zhao

**Affiliations:** ^1^ Department of Neurosurgery, The First Affiliated Hospital of Harbin Medical University, Nangang District, Harbin, Heilongjiang Province, 150001, People's Republic of China; ^2^ Institute of Brain Science, Harbin Medical University, Nangang District, Harbin, Heilongjiang Province, 150001, People's Republic of China; ^3^ College of Bioinformatics Science and Technology, Harbin Medical University, Nangang District, Harbin, Heilongjiang Province, 150081, People's Republic of China

**Keywords:** long noncoding RNA (lncRNA), urothelial cancer associated 1 (UCA1), cancer, prognosis

## Abstract

Urothelial cancer associated 1 (UCA1) as an oncogenic long non-coding RNA (LncRNA) was aberrantly upregulated in various solid tumors. Numerous studies have demonstrated overexpression of UCA1 is an unfavorable prognostic indicator in cancer patients. This study aimed to further explore the prognosis role and clinical significance of UCA1 in cancer. Eligible studies were recruited by a systematic search in PubMed, Embase, Cochrane Library and Web of Science databases. A total of 19/16 studies with 1587/1291 cancer patients were included to evaluate the association between UCA1 expression and overall survival (OS) and clinicopathological factors of malignancies by computing hazard ratio (HR), odds ratios (OR) and confidence interval (CI). The meta-analysis indicated overexpression of UCA1 was significantly correlated with unexpected OS in patients with cancer (pooled HR = 1.85, 95% CI 1.62–2.10, *p* < 0.001). There was also a significantly negative association between high level of UCA1 and poor grade cancer (pooled OR = 2.74, 95% CI 2.04–3.70, *p* < 0.001) and positive lymphatic metastasis (pooled OR = 2.43, 95% CI 1.72–3.41, *p* < 0.001). In conclusion, our study suggested that UCA1 was correlated with more advanced clinicopathological features and poor prognosis as a novel predictive biomarker of patients with various tumors.

## INTRODUCTION

Non-coding RNAs (ncRNAs) encoded by the eukaryotic genome are considered as a large number of RNAs which are not translated into proteins. Indeed, more than 90% of noncoding RNAs used to be recognized as “biological noises” in transcription progression compared with about 3% protein-coding genes until the development of high-throughput sequencing technology and large-scale mapping of transcriptomes [1, 2]. Based on structural features and biological functions, ncRNAs family has been further classified into housekeeping and regulatory ncRNAs. Regulatory ncRNAs upon nucleotide length are generally divided into two subgroups: (1) short ncRNAs shorter than 200 nucleotides such as microRNAs (miRNAs); (2) long noncoding RNAs (lncRNAs) longer than 200 nucleotides such as intergenic lncRNAs (lincRNAs) and circular RNAs (circRNAs) [3, 4]. Accumulating evidence have revealed the major transcriptional and post-transcriptional regulation roles of lncRNAs emerge in transcription factor recruitment, chromatin remodeling, histone modification, pre-mRNA splicing, molecular sponge and scaffold, which are involved in development of normal tissue or organ and carcinogenesis and aggression of diverse malignancies [5–8].

Urothelial cancer associated 1 also known as UCA1 is located on 19p13.12 encodes 3 isoforms (1.4, 2.2 and 2.7 kb) with ployA tails, in which the 2.2 kb isoform is called cancer upregulated drug resistant (CUDR). Previous study has shown UCA1, a highly conserved nuclear-enriched lncRNA, is ubiquitous in different development stages of urinary system, productive system, digestive system and respiratory system [9]. In cancers, UCA1 as an oncogene exhibited regulatory mechanisms responsible for cell proliferation, invasion, metastasis, apoptosis, metabolism and chemoresistance [10]. Cheng et al. reported that downregulation of UCA1 impaired chemoresistance of non-small cell lung cancer cells with non-T790M to gefitinib by inhibition of the AKT/mTOR pathway [11]. Na et al. indicated knockdown of UCA1 inhibited cell proliferation and induced cell apoptosis by inactivation of KLF4-KRT6/13 cascade in prostate cancer [12]. Wang et al. showed that UCA1 as an endogenous sponge restored the negative effect of miR-216b on the growth and metastasis of hepatocellular carcinoma cells through activating FGFR1/ERK signaling pathway [13]. Hu et al. revealed the X protein encoded by HBV increased UCA1 expression which inhibited cell apoptosis and promoted cell proliferation and carcinogenesis by HBx-UCA1/EZH2-p27Kip1 signaling axis in hepatocellular carcinoma cells [14].

Furthermore, increasing interest has been focused on whether UCA1 acts as a diagnosis and prognosis biomarker in cancer detection and treatment. UCA1 was firstly characterized as an effective diagnosis biomarker in bladder cancer with a high sensitivity and specificity (80.9% and 91.8%) [15]. Meanwhile, growing subsequent evidence suggest that aberrant overexpression of UCA1 is associated with high risk of poor outcome or clinicopathological characteristics in breast cancer, colorectal cancer, bladder cancer, esophageal squamous cell carcinoma, epithelial ovarian cancer, gastric cancer, hepatocellular carcinoma, melanoma, non-small cell lung cancer, prostate cancer and tongue squamous cell carcinoma [11–13, 15–27]. Thus, it is necessary to certificate the potential correlation between UCA1 expression and malignancies by a comprehensive analysis. In this meta-analysis, we qualified and evaluated present studies to explore the association of UCA1 with prognostic and clinicopathological significance in patients with different types of carcinomas.

## RESULTS

### Study selection and characteristics

**Figure 1:**
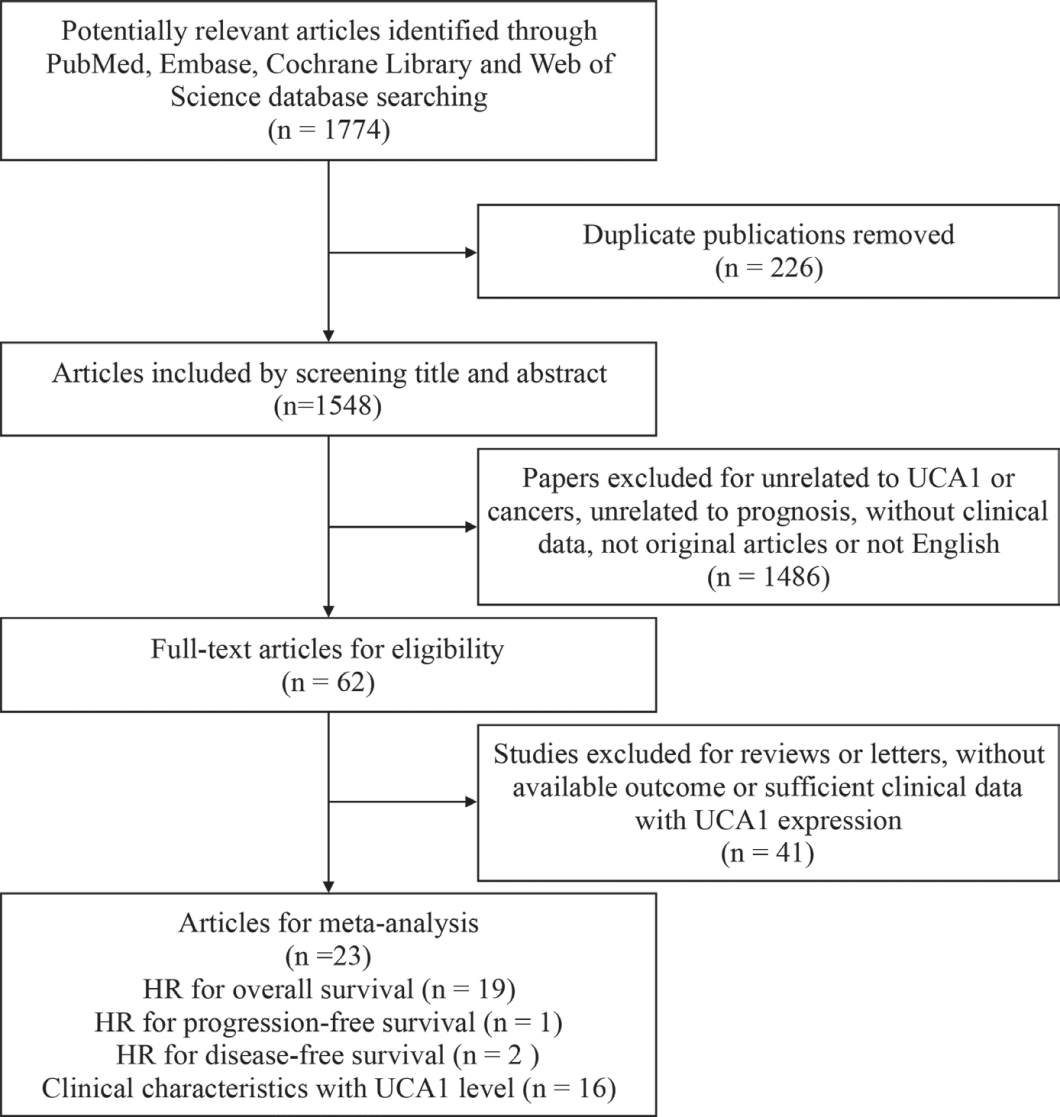
Workflow of searching strategy and study selection in the meta-analysis

Upon an electrical search on PubMed, Embase, Cochrane Library and Web of Science databases, a total of 19 eligible OS papers including 1587 tissue specimens from 1774 records published from 2014 to 2017 were enrolled by a cautious searching strategy and full-text screening, which were based on inclusion and exclusion criteria in this meta-analysis (Figure 1). Participants in 19 OS studies were all Asian with 10 types of tumors including non-small cell lung carcinoma (NSCLC), gastric cancer (GC), colorectal cancer (CRC), esophageal squamous cell carcinoma (ESCC), prostate cancer (PC), hepatocellular cancer (HCC), epithelial ovarian cancer (EOC), endometrial cancer (EC), pancreatic cancer (PAC), and breast cancer (BC) (Supplementary Table 1). The main characteristics of retrieved OS studies were summarized in Table 1. Meanwhile, total 16 available clinicopathological studies containing 1291 tissue samples were collected to analyze the correlation between UCA1 level and clinicopathological data (clinical stage, tumor size, lymphatic and distant metastasis) shown in (Supplementary Table 2). And all clinicopathological studies were Asian with 8 kinds of tumors above except prostate and breast cancer. Quality evaluation based on reporting recommendations for tumor marker prognostic studies (REMARK) guideline reflected quality score ranged from 40% to 80% in (Supplementary Table 3). With inconsistent cut-off values due to different detection methods, the patients were separated into high and low level of UCA1 groups. HRs with 95% CIs were extracted from multivariate analysis in 14 studies, univariate analysis in 5 studies (estimated effects collected from Kaplan–Meier survival curve in 3 studies).

**Table 1:** The main characteristics of included OS studies in the prognosis based on meta-analysis

Study	Year	Region	Tumor type	Sample size	Specimen	Method	Cut-off value	Outcome	Analysis	Quality score (%)
Li, et al.^20^	2014	China	ESCC	90	Tissue	qRT-PCR	Mean (NG)	OS	Multivariable	75
Zheng, et al.^22^	2015	China	GC	112	Tissue	qRT-PCR	Median (17.24)	OS	Multivariable	75
Wang, et al.^13^	2015	China	NSCLC	60	Tissue	qRT-PCR	Median (NG)	OS	Multivariable	75
Ni, et al.^17^	2015	China	CRC	54	Tissue	qRT-PCR	Median (NG)	OS	Univariable analysis	60
Na, et al.^12^	2015	China	PC	40	Tissue	qRT-PCR	Median (NG)	OS	Univariable	40
Gao, et al.^23^	2015	China	GC	20	Tissue	qRT-PCR	NG	OS	Multivariable	40
Nie, et al.^26^	2015	China	NSCLC	112	Tissue	qRT-PCR	Youden index (NG)	OS	Multivariable	70
Tao, et al.^43^	2015	China	CRC	80	Tissue	qRT-PCR	Upper quartile (NG)	OS	Multivariable	70
Wang, et al.^13^	2015	China	HCC	98	Tissue	qRT-PCR	Median (NG)	OS	Multivariable	70
Yang, et al.^44^	2015	Korea	HCC	240	Tissue	microarray	Median (6.51)	OS	Univariable analysis	50
Bian, et al.^19^	2016	China	CRC	90	Tissue	qRT-PCR	Median (NG)	OS	Multivariable	70
Yang, et al.^21^	2016	China	EOC	53	Tissue	qRT-PCR	Median (NG)	OS	Multivariable	75
Zhang, et al.^45^	2016	China	EOC	117	Tissue	qRT-PCR	Median (NG)	OS	Multivariable	70
Lu, et al.^46^	2016	China	EC	45	Tissue	qRT-PCR	Median (NG)	OS	Univariable	65
Shang, et al.^24^	2016	China	GC	77	Tissue	qRT-PCR	Median (NG)	OS	Multivariable	75
Chen, et al.^47^	2016	China	PAC	128	Tissue	qRT-PCR	Mean (NG)	OS	Multivariable	80
Fu, et al.^48^	2016	China	PAC	80	Tissue	qRT-PCR	Median (NG)	OS	Multivariable	80
Liu, et al.^49^	2016	China	BC	54	Tissue	qRT-PCR	Median (NG)	OS	Univariable	45
Zuo, et al.^50^	2017	China	GC	37	Tissue	qRT-PCR	Median (NG)	OS	Multivariable	75

Abbreviations: ESCC, esophageal squamous cell carcinoma; GC, gastric cancer; NSCLC, non-small cell lung carcinoma; CRC, colorectal cancer; PC, prostate cancer; HCC, hepatocellular cancer; EOC, epithelial ovarian cancer; EC, endometrial cancer; PAC, pancreatic cancer; BC, breast cancer; qRT-PCR, quantitative real-time PCR; OS, overall survival; NG, not given.

### Association of UCA1 expression with overall survival in human cancers

**Figure 2:**
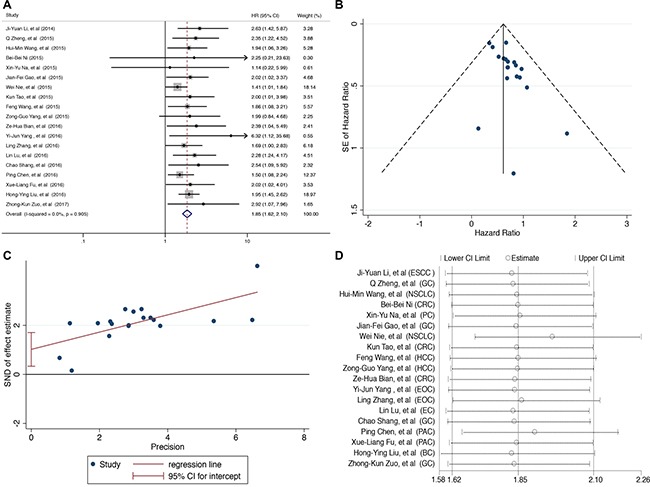
Prognostic value of UCA1 for OS of cancer patients (**A**) Forest plot of HR studies of UCA1 for OS in a fixed effect model. Each study is represented by a square and the center of which denotes the HR with a horizontal 95% Cis lines. The diamond shows the overall HR for combined results. Weights are from a fixed effect analysis. (**B**) Funnel plot for potential publication bias in OS analysis. Standard error (SE) of hazard ratio displays a measure of study size on the vertical axis against hazard ratio on the horizontal axis. (**C**) Egger's test for potential publication bias in OS analysis. Standard normal deviate (SND) is defined as the hazard ratio divided by its standard error which is regressed against the estimate's precision. (**D**) Sensitivity analysis of the effect of the individual study on the pooled HRs.

To assess the association between the UCA1 expression and OS of all cancer patients, a total of 1587 patients with HRs and 95% CIs were included. A significant association was observed between high UCA1 level and poor OS in patients with all 10 types of cancer (pooled HR = 1.85, 95% CI 1.62–2.10, *p* < 0.001) and no obvious heterogeneity existed across 19 studies under a fixed effect model (I^2^ = 0.0%, p_H_ = 0.905) in Figure 2A.

The subgroup analysis in a fixed effect model, random effect model and meta-regression was conducted by cancer types, analysis methods, sample sizes and quality scores for a subsequent investigation of potential heterogeneity in Table 2. It revealed a significant association between increased UCA1 and OS in patients with GC (pooled HR = 2.32, 95% CI 1.61–3.33, *p* < 0.001), CRC (pooled HR = 2.16, 95% CI 1.29–3.62, *p* = 0.004), HCC (pooled HR = 1.89, 95% CI 1.20–3.00, *p* = 0.007), and PAC (pooled HR = 1.60, 95% CI 1.16–2.22, *p* = 0.004). There showed no statistical significance of heterogeneity test in subgroups of GC (I^2^ = 0.0%, p_H_ = 0.928), CRC (I^2^ = 0.0%, p_H_ = 0.948), HCC (I^2^ = 0.0%, p_H_ = 0.899), and PAC (I^2^ = 0.0%, p_H_ = 0.457). Then, a significant effect of elevated UCA1 on OS emerged in multivariable analysis subgroup (pooled HR = 1.80, 95% CI 1.55–2.09, *p* < 0.001) and univariable analysis subgroup (pooled HR = 1.98, 95% CI 1.55–2.55, *p* < 0.001). No statistically significant heterogeneity was in multivariable analysis subgroup (I^2^ = 0.0%, p_H_ = 0.722) and univariable analysis subgroup (I^2^ = 0.0%, p_H_ = 0.956). Meanwhile, we also detected a significant correlation between overexpression of UCA1 and poor prognosis of cancer patients in large specimen size subgroup (pooled HR = 1.68, 95% CI 1.41–2.00, *p* < 0.001) and small specimen size subgroup (pooled HR = 2.07, 95% CI 1.71–2.50, *p* < 0.001) with no evident heterogeneity (I^2^ = 0.0%, p_H_ = 0.639 and I^2^ = 0.0%, p_H_ = 0.979) respectively. To the quality score subgroups, we obtained similar association of upregulation of UCA1 with OS in high quality score subgroup (pooled HR = 1.98, 95% CI 1.58–2.48, *p* < 0.001) and low quality score subgroup (pooled HR = 1.79, 95% CI 1.53–2.09, *p* < 0.001). No evidence of significant heterogeneity was found across 2 subgroups (I^2^ = 0.0%, p_H_ = 0.576 and I^2^ = 0.0%, p_H_ = 0.921). Quantified heterogeneous test by meta-regression indicated that no specific factor accounted for the heterogeneity among studies of interest consistent with the outcomes of subgroup analysis in cancer type, analysis method, tumor sample size and quality score covariates.

**Table 2:** Subgroup and meta-regression analysis of HRs in different cancer type, analysis method, sample size and quality score subgroup

Subgroup analysis	No. of studies	No. of patients	Pool HR (95% CI)		Meta-regression (*p* value)	Heterogeneity (random)	
			Fixed	Random		I^2^(%)	*p* value
Overall survival	19	1587	1.85 (1.62–2.10)	1.85 (1.62–2.10)	-	0.0	0.905
Cancer type							
Digestive system	12	1106	1.97 (1.63–2.38)	1.97 (1.63–2.38)	-	0.0	0.963
GC	4	246	2.32 (1.61–3.33)	2.32 (1.61–3.33)	0.987	0.0	0.928
CRC	3	224	2.16 (1.29–3.62)	2.16 (1.29–3.62)	0.817	0.0	0.948
HCC	2	338	1.89 (1.20–3.00)	1.89 (1.20–3.00)	0.925	0.0	0.899
PAC	2	208	1.60 (1.16–2.22)	1.60 (1.16–2.22)	0.240	0.0	0.457
ESCC	1	90	2.63 (1.29–5.35)	2.63 (1.29–5.35)	-	-	-
Respiratory system	2	172	1.51 (1.16–1.98)	1.51 (1.16–1.98)	-	0.0	0.328
NSCLC	2	172	1.51 (1.16–1.98)	1.51 (1.16–1.98)	0.454	0.0	0.328
Reproductive system	4	269	1.98 (1.57–2.50)	1.98 (1.57–2.50)	-	0.0	0.511
EOC	2	170	1.88 (1.15–3.09)	2.50 (0.77–8.13)	0.883	51.2	0.152
EC	1	45	2.28 (1.24–4.18)	2.28 (1.24–4.18)	0.961	-	-
BC	1	54	1.95 (1.45–2.62)	1.95 (1.45–2.62)	0.795	-	-
Urinary system	1	40	1.14 (0.22–5.95)	1.14 (0.22–5.95)	-	-	-
PC	1	40	1.14 (0.22–5.95)	1.14 (0.22–5.95)	0.530	-	-
Analysis method							
Multivariable analysis	14	1154	1.80 (1.55–2.09)	1.80 (1.55–2.09)	-	0.0	0.722
Univariable analysis	5	433	1.98 (1.55–2.55)	1.98 (1.55–2.55)	0.913	0.0	0.956
Sample size							
Size ≥ 90	8	987	1.68 (1.41–2.00)	1.68 (1.41–2.00)	-	0.0	0.639
Size < 90	11	600	2.07 (1.71–2.50)	2.07 (1.71–2.50)	0.538	0.0	0.979
Quality scores							
Score ≥ 75	8	637	1.98 (1.58–2.48)	1.98 (1.58–2.48)	-	0.0	0.576
Score < 75	11	950	1.79 (1.53–2.09)	1.79 (1.53–2.09)	0.296	0.0	0.921

Abbreviations: ESCC, esophageal squamous cell carcinoma; GC, gastric cancer; NSCLC, non-small cell lung carcinoma; CRC, colorectal cancer; PC, prostate cancer; HCC, hepatocellular cancer; EOC, epithelial ovarian cancer; EC, endometrial cancer; PAC, pancreatic cancer; BC, breast cancer.

Publication bias evaluated by funnel plot and Egger's test indicated that there was evident asymmetry in this meta-analysis (p_Egger’s_ = 0.006, Figure 2B and 2C). And publication bias of subgroup showed small-studies effects existed in large tumor sample size (p_Egger’s_ = 0.001), high quality score (p_Egger’s_ < 0.001) and multivariate analysis subgroup (p_Egger’s_ < 0.001) in (Supplementary Table 4). However, sensitivity analysis by removing each research in turn showed the residual pooled HRs of OS were not impacted dramatically in Figure 2D.

### Correlation between UCA1 level and clinical characteristics in patients with cancer

In the clinicopathological studies, 16 researches consisting of 1291 tumor samples with a correlation between clinicopathological features and UCA1 expression were retrieved in OR analysis. Clinical stage, lymph node metastasis, distant metastasis and tumor size data were collected to analyze. UCA1 expression was significantly different in these clinicopathological factors (Supplementary Table 2). We found high UCA1 expression was associated with high grade cancer (pooled OR = 2.74, 95% CI 2.04–3.70, *p* < 0.001), positive lymphatic metastasis (pooled OR = 2.43, 95% CI 1.72–3.41, *p* < 0.001), and distant metastasis (pooled OR = 2.10, 95% CI 1.13–3.89, *p* < 0.001) in Table 3, 4, 5, 6.

**Table 3:** The subgroup and meta-regression analysis of the association and heterogeneity between high UCA1 expression and clinical stage

Subgroup analysis	No. of studies	No. of patients	Pool OR (95% CI)		Meta-regression (*p* value)	Heterogeneity (random)	
			Fixed	Random		I^2^(%)	*p* value
ORs of tumor stage subgroup	16	1291	2.63 (2.07–3.33)	2.74 (2.04–3.70)	-	30.8	0.116
Cancer type							
Digestive system	11	909	2.45 (1.86–3.24)	2.61 (1.74–3.91)	-	47.4	0.04
GC	2	149	3.32 (1.66–6.66)	4.18 (1.10–16.0)	0.855	61.2	0.109
CRC	4	304	2.23 (1.38–3.60)	2.38 (1.18–4.80)	0.616	49.5	0.114
HCC	2	158	2.78 (1.46–5.30)	2.53 (0.56–11.4)	0.702	79.9	0.026
PAC	2	208	1.68 (0.91–3.09)	1.97 (0.66–5.89)	0.569	54.2	0.140
ESCC	1	90	3.64 (1.49–8.88)	3.64 (1.49–8.88)	0.851	-	-
Respiratory system	2	172	3.74 (1.82–7.70)	3.71 (1.79–7.69)	-	0.0	0.368
NSCLC	2	172	3.74 (1.82–7.70)	3.71 (1.79–7.69)	0.869	0.0	0.368
Reproductive system	3	210	2.86 (1.60–5.14)	2.88 (1.60–5.17)	-	0.0	0.730
EOC	2	168	2.66 (1.42–5.00)	2.66 (1.42–5.00)	0.704	0.0	0.648
EC	1	42	4.64 (0.98–22.0)	4.64 (0.98–22.0)	-	-	-
Sample size							
Size ≥ 90	7	745	2.50 (1.84–3.40)	2.53 (1.80–3.55)	-	15.7	0.310
Size < 90	9	546	2.83 (1.94–4.12)	3.12 (1.84–5.29)	0.761	44.2	0.074
Quality scores							
Score ≥ 75	7	560	2.79 (1.94–4.00)	3.07 (1.89–5.00)	-	37.8	0.141
Score < 75	9	731	2.51 (1.83–3.45)	2.56 (1.72–3.82)	0.768	32.8	0.156

Abbreviations: ESCC, esophageal squamous cell carcinoma; GC, gastric cancer; NSCLC, non-small cell lung carcinoma; CRC, colorectal cancer; HCC, hepatocellular cancer; EOC, epithelial ovarian cancer; EC, endometrial cancer; PAC, pancreatic cancer.

**Table 4:** The subgroup and meta-regression analysis of the association and heterogeneity between high UCA1 expression and tumor size

Subgroup analysis	No. of studies	No. of patients	Pool OR (95% CI)		Meta-regression (*p* value)	Heterogeneity (random)	
			Fixed	Random		I^2^(%)	*p* value
ORs of tumor size subgroup	13	1090	1.47 (1.15–1.87)	1.45 (0.99–2.14)	-	57.0	0.006
Cancer type							
Digestive system	9	762	1.41 (1.06–1.88)	1.42 (0.66–2.37)	-	64.6	0.004
GC	2	149	0.71 (0.37–1.35)	1.01 (0.17–6.04)	0.603	81.5	0.02
CRC	3	250	1.89 (1.11–3.22)	1.81 (0.67–4.90)	0.822	69.5	0.038
HCC	2	155	1.57 (0.83–2.96)	1.47 (0.55–3.93)	0.667	55.2	0.135
PAC	2	208	1.58 (0.92–2.73)	1.49 (0.60–3.69)	-	61.3	0.108
Respiratory system	2	172	1.98 (1.04–3.76)	1.99 (1.04–3.78)	-	0.0	0.350
NSCLC	2	172	1.98 (1.04–3.76)	1.99 (1.04–3.78)	0.938	0.0	0.350
Reproductive system	2	156	1.36 (0.73–2.54)	1.19 (0.34–4.18)	-	71.6	0.060
EOC	2	156	1.36 (0.73–2.54)	1.19 (0.34–4.18)	0.623	71.6	0.060
Sample size							
Size ≥ 90	6	643	1.76 (1.28–2.41)	1.83 (1.02–3.28)	-	69.1	0.006
Size < 90	7	447	1.13 (0.77–1.66)	1.13 (0.71–1.80)	0.575	29.5	0.203
Quality scores							
Score ≥ 75	6	470	1.07 (0.75–1.54)	1.08 (0.58–2.00)	-	61.2	0.024
Score < 75	7	620	1.92 (1.38–2.68)	1.88 (1.23–2.89)	0.599	37.9	0.140

Abbreviations: GC, gastric cancer; NSCLC, non-small cell lung carcinoma; CRC, colorectal cancer; HCC, hepatocellular cancer; EOC, epithelial ovarian cancer; PAC, pancreatic cancer.

**Table 5:** The subgroup and meta-regression analysis of the association and heterogeneity between high UCA1 expression and lymph node metastasis

Subgroup analysis	No. of studies	No. of patients	Pool OR (95% CI)		Meta-regression (*p* value)	Heterogeneity (random)	
			Fixed	Random		I^2^(%)	*p* value
ORs of lymph node metastasis subgroup	15	1229	2.26 (1.78–2.86)	2.43 (1.72–3.41)	-	47.1	0.023
Cancer type							
Digestive system	10	849	2.05 (1.55–2.71)	2.17 (1.42–3.31)	-	52.1	0.027
GC	2	149	1.36 (0.71–2.60)	1.99 (0.32–12.5)	0.290	81.0	0.022
CRC	4	304	2.07 (1.28–3.34)	2.07 (1.25–3.43)	0.290	5.6	0.365
HCC	1	98	5.46 (2.26–13.2)	5.46 (2.26–13.2)	0.970	-	-
PAC	2	208	1.48 (0.85–2.57)	1.46 (0.78–2.73)	0.235	19.6	0.265
ESCC	1	90	3.57 (1.48–8.74)	3.57 (1.48–8.74)	0.595	-	-
Respiratory system	2	172	2.23 (1.17–4.25)	2.54 (0.70–9.23)	-	71.1	0.063
NSCLC	2	172	2.23 (1.17–4.25)	2.54 (0.70–9.23)	0.372	71.1	0.063
Reproductive system	3	208	3.65 (1.96–6.81)	3.70 (1.98–6.91)	-	0.0	0.509
EOC	2	163	3.16 (1.59–6.27)	3.16 (1.59–6.27)	0.495	0.0	0.684
EC	1	45	7.84 (1.77–34.8)	7.84 (1.77–34.8)	0.961	-	-
Sample size							
Size ≥ 90	7	740	2.09 (1.55–2.83)	2.14 (1.36–3.53)	-	52.5	0.049
Size < 90	8	489	2.55 (1.74–3.73)	2.89 (1.67–5.00)	0.589	46.8	0.068
Quality scores							
Score ≥ 75	7	560	2.02 (1.43–2.84)	2.27 (1.29–4.00)	-	58.9	0.024
Score < 75	8	669	2.51 (1.81–3.49)	2.60 (1.69–4.00)	0.597	36.9	0.135

Abbreviations: ESCC, esophageal squamous cell carcinoma; GC, gastric cancer; NSCLC, non-small cell lung carcinoma; CRC, colorectal cancer; HCC, hepatocellular cancer; EOC, epithelial ovarian cancer; EC, endometrial cancer; PAC, pancreatic cancer.

**Table 6:** The subgroup and meta-regression analysis of the association and heterogeneity between high UCA1 expression and distant metastasis

Subgroup analysis	No. of studies	No. of patients	Pool OR (95% CI)		Meta-regression (*p* value)	Heterogeneity (random)	
			Fixed	Random		I^2^(%)	*p* value
ORs of distant metastasis subgroup	8	687	2.05 (1.42–2.96)	2.10 (1.13–3.89)	-	56.5	0.024
Cancer type							
Digestive system	7	642	1.89 (1.29–2.78)	1.84 (0.97–3.49)	-	55.7	0.035
GC	1	112	6.25 (1.48–26.4)	6.25 (1.48–26.4)	-	-	-
CRC	3	224	2.08 (0.97–4.48)	1.98 (0.85–4.59)	-	8.0	0.337
HCC	1	98	5.46 (2.26–13.2)	5.46 (2.26–13.2)	-	-	-
PAC	2	208	1.66 (0.86–3.21)	1.66 (0.85–3.22)	-	0.0	0.446
Reproductive system	1	45	6.25 (1.48–26.4)	6.25 (1.48–26.4)	-	-	-
EC	1	45	6.25 (1.48–26.4)	6.25 (1.48–26.4)	-	-	-
Sample size							
Size ≥ 90	4	428	1.98 (1.28–3.07)	1.96 (0.81–4.75)	-	71.9	0.014
Size < 90	4	259	2.22 (1.12–4.42)	2.34 (0.86–6.37)	0.752	44.3	0.143
Quality scores							
Score ≥ 75	3	320	1.20 (0.71–2.10)	1.14 (0.55–2.36)	-	38.4	0.197
Score < 75	11	367	3.43 (2.01–5.85)	3.38 (1.69–6.74)	0.104	31.4	0.212

Abbreviations: GC, gastric cancer; CRC, colorectal cancer; HCC, hepatocellular cancer; EC, endometrial cancer; PAC, pancreatic cancer.

The subgroup analysis stratified by cancer type, sample size and quality score uncovered the resource of heterogeneity under the fixed effect model, random effect model and meta-regression analysis (Table 3, 4, 5, 6). There existed a significantly statistical heterogeneity across distant metastasis subgroup (I^2^ = 56.5%, p_H_ = 0.024) in 8 clinicopathological studies shown in Table 6. In distant metastasis heterogeneity analysis, we detected a significant heterogeneity in large sample size (I^2^ = 71.9%, p_H_ = 0.014) subgroup. No evidence of statistical heterogeneity was detected in cancer type and lymphatic metastasis subgroup (I^2^ = 30.8%, p_H_ = 0.116 and I^2^ = 47.1%, p_H_ = 0.023, respectively). However, only CRC showed clinical significance and no obvious heterogeneity (pooled OR = 2.38, 95% CI 1.18–4.80, *p* < 0.001, I^2^ = 49.5%, p_H_ = 0.114) by stratification analysis of cancer type. In subsequent stratification analysis of lymphatic metastasis, there was no evidence of significant heterogeneity in CRC (pooled OR = 2.07, 95% CI 1.25–3.43, *p* = 0.003, I^2^ = 5.6%, p_H_ = 0.365) and EOC (pooled OR = 3.16, 95% CI 1.59–6.27, p *p* < 0.001, I^2^ = 0.0%, p_H_ = 0.684) subgroup.

A Egger's linear regression test was conducted to evaluate publication bias of these clinicopathological covariates. The result showed no publication bias existed between tumor size and distant metastasis subgroup in (Supplementary Table 5) (p_Egger’s_ = 0.622 and p_Egger’s_ = 0.653, respectively) and no asymmetry among 4 subgroups in the sensitivity analysis (Supplementary Figure 1).

## DISCUSSION

Cancer attacks all humankinds as a major cause of morbidity and mortality in worldwide regions with approximately 14.1 million new cancer cases and 8.2 million cancer-related deaths in 2012 [28]. Despite significant advances in cancer treatment, an unexpected long-term overall survival is still an important public health challenge. Therefore, novel strategies for detection of diagnostic and prognostic biomarkers and identification of potential therapeutic targets have attracted increasing interest.

The emerging next generation sequencing technology and large-scale of transcriptome mapping have provided an opportunity to facilitate to understand genome information and identify over 90% non-coding RNAs regarded as “transcriptional noise” before. Continuing advances in these new technologies indicate the vital and complex functions of lncRNAs in gene regulation. With these updated views, the central dogma may be rewritten [6, 7]. Recent numerous studies have confirmed that lncRNAs as oncogenes or tumor suppressors play important regulatory roles in biological progress of a broad range of cancers or other human diseases. Substantial progress of lncRNAs demonstrate they may act as molecular scaffolds, sponges or co-activators by interaction with DNA, RNA or proteins in cancer nosogenesis [1, 5, 6]. Currently, a growing body of evidence revealed aberrant UCA1 as an oncogene in various malignancies [11–13, 15–27]. Intensive researches have showed overexpression of UCA1 can promote the progression of proliferation, invasion, migration, metastasis, chemoresistance in a variety of cancers [10]. Moreover, the dysregulation of UCA1 was also found in acute myocardial infarction, kidney damage and neurodegenerative diseases [10]. In cancer, the binding between UCA1 promoter core region and transcription factors or complex (C/EBPα, Ets-2, TAZ/YAP/TEAD and SMAD2/3) can enhance UCA1 promoter activity and gene expression [29, 30]. The upregulation of UCA1 is responsible for tumor cell proliferation by suppressing G0/G1 cell cycle arrest and apoptosis. Furthermore, increased UCA1 promotes cancer invasion and metastasis by activation of metastasis genes including MMP14, FGFR1/ERK and ZEB1/2-FSCN1, and enhances chemoresistance by a set of anti-apoptosis genes and signaling pathways (PARP/BCL-2, CREB1/BCL-2/RAB22A, AKT/mTOR and Wnt signaling pathway) [11, 13, 19, 21, 24, 31–33]. UCA1 is also a key molecular sponge or competing endogenous RNA (ceRNA) for miR-1, miR-204–5p, miR-193a-3p, miR-145 and miR-216 [13, 19, 26, 32, 34]. Simultaneously, UCA1 is associated with clinical parameters and prognosis of cancer patients and may be a potential diagnosis biomarker in gastric cancer, hepatocellular cancer and bladder cancer [15, 22, 23, 35].

Here, we performed this meta-analysis to explore the correlation between high expression of UCA1 and clinicopathological characteristics and evaluate the prognosis role of UCA1 for cancer patients. All of these results above suggest that high UCA1 expression may be regarded as an unfavorable predictor in different cancers (pooled HR = 1.85, 95% CI 1.62–2.10, *p* < 0.001). Meanwhile, the pooled data of eligible studies also indicated that high UCA1 expression was significant correlated with poor grade cancer (pooled OR = 2.74, 95% CI 2.04–3.70, *p* < 0.001, I^2^ = 49.5%, p_H_ = 0.1) and positive lymph node metastasis (pooled OR = 2.43, 95% CI 1.72–3.41, *p* < 0.001).

Otherwise, it should be acknowledged that several limitations existed in this current meta-analysis. First, almost all available studies were performed in China. The prognostic role of UCA1 should be taken cautiously in other regions and ethnicities. Second, inadequate data from some types of cancer and subgroup analysis in HRs and ORs may be the origin of heterogeneity. Third, the inconsistent cut-off value of UCA1 expression due to different methods and criteria may result in some heterogeneity. Finally, included papers were only English and most of which reported positive results, which may generate publication bias. Thus, the predictive significance of evaluated UCA1 in poor prognosis of patients with cancer might be overestimated to some extent. In addition, recent publications demonstrated a friendly and open user server by computational approaches will facilitate novel technologies and findings accessible to the public and enhance their impacts [36–38]. Hence, we appeal to establish a web-server of raw and integrated data for further analysis in drug design and in-depth investigation, such as UCA1. In conclusion, the present analysis showed that overexpression of UCA1 might predict a poor prognosis in various types of malignancies, especially in Chinese population and was associated with poor cancer stage and positive lymphatic metastasis. Therefore, UCA1 may serve as an effective biomarker to predict prognosis and tumor progression of patients with cancer. Nevertheless, more large-scale and well-designed studies are required to update the findings of this analysis.

## MATERIALS AND METHODS

### Search strategy and study selection criteria

Systematic literature searches were conducted in PubMed, Embase, Cochrane Library and Web of Science databases (up to January 17, 2017) with the following searching strategy: “UCA1” OR “UCA 1” OR “UCA-1” OR “urothelial cancer associated 1” OR “urothelial cancer associated-1” OR “CUDR” OR “LINC00178” OR “NCRNA00178” OR “onco-lncRNA-36” OR “ENSG00000214049”) AND (“cancer” OR “tumor” OR “tumour” OR “carcinoma” OR “neoplasm” OR “adenoma” OR “sarcoma”). We manually searched retrieved references for potentially missing literatures. The cited articles were excluded from duplicated firstly, then titles and abstracts were carefully scanned to eliminate irrelevant studies. Finally, we prudently reviewed full texts of potential retrieved studies. Studies were available upon the eligibility criteria: (1) showed the relationship between the dichotomous UCA1 levels and prognosis and clinicopathological characteristics of patients with any type of cancer; (2) calculated HRs and 95% CIs for overall survival (OS), progression-free survival (PFS), disease-free survival (DFS); (3) were published in English. Duplicated, non-dichotomous expression of UCA1 or non-English articles, absence of key survival outcome such as HRs, 95% CIs or Kaplan-Meier survival curve, reviews, letters, laboratory studies of non-human research or comments were omitted.

### Data extraction

Eligible data were deliberatively judged and double checked from available studies based on criteria of inclusion and exclusion. To each study, we carefully extracted the following information: first author, journal, year of publication, country, ethnicity of the study population, type of specimen, carcinoma type, number of patients, detection method, cut-off value, follow-up, quality score and HR and 95% CI for OS, PFS, or DFS. Quality assessment of the available studies was performed upon the REMARK guideline [39]. HR, as a dominant indicator of interest, was respectively extracted from multivariable analysis, univariate analysis, additional information of first authors’ e-mails and estimated from graphical survival plots by Engauge Digitizer 4.1 software as described previously, if only Kaplan-Meier curve existed [40, 41]. Clinical parameters with dichotomous UCA1 levels also were retrieved such as clinical stage (TNM stage), tumor size, lymphatic and distant metastasis.

### Statistical analysis

All analysis was conducted with STATA software version 12.0 (STATA Corporation, College Station, TX, USA). Odds Ratios (ORs) were performed to analyze the association of UCA1 expression with clinical stage, tumor size, lymphatic and distant metastasis and HRs with corresponding 95% CIs were utilized to estimate the relationship strength between UCA1 expression and patients’ prognosis. If the HRs were not directly reported in original articles, we calculated the essential data upon the previously reported methods [40]. The pooled HRs were estimated using a fixed effect model (Mantel-Haenszel) in the absence of heterogeneity, if heterogeneity was observed applying a random effect model (DerSimoian-Laird) and meta-regression analysis [41, 42]. The heterogeneity tests of combined HRs and ORs were carried out by Cochran's *Q* test and Higgins I-squared statistic (P_H_ < 0.1 and I^2^ > 50%). To further explore the potential heterogeneity factors and outcome stability in studies, a subgroup analysis and a sensitivity analysis were utilized respectively in the meta-analysis. Publication bias was evaluated by a funnel plot and Egger's linear regression test with a significant publication bias by *P* < 0.05.

## SUPPLEMENTARY MATERIALS FIGURES AND TABLES









## Abbreviations

ncRNAs: non-coding RNAs
LncRNA: long non-coding RNA
ncRNAs: non-coding RNAs
OS: overall survival
PFS: progression-free survival
DFS: disease-free survival
HR: hazard ratio
OR: odds ratios
CI: confidence interval
miRNAs: microRNAs
lincRNAs: intergenic lncRNAs
circRNAs: circular RNAs
NSCLC: non-small cell lung carcinoma
GC: gastric cancer
CRC: colorectal cancer
ESCC: esophageal squamous cell carcinoma
PC: prostate cancer
HCC: hepatocellular cancer
EOC: epithelial ovarian cancer
EC: endometrial cancer
PAC: pancreatic cancer
BC: breast cancer
REMARK: reporting recommendations for tumor marker prognostic studies
