# Supplementary material for: Prognostic and clinicopathological role of long non-coding RNA UCA1 in various carcinomas
Source: Oncotarget. 2017 Mar 9;8(17):28373–84. doi: 10.18632/oncotarget.16059 (PMC5438656; doi:10.18632/oncotarget.16059)
Supplement: Supplementary file 1 [file oncotarget-08-28373-s001.pdf]

Prognostic and clinicopathological role of long non-coding RNA UCA1 in various carcinomas

Supplementary Materials

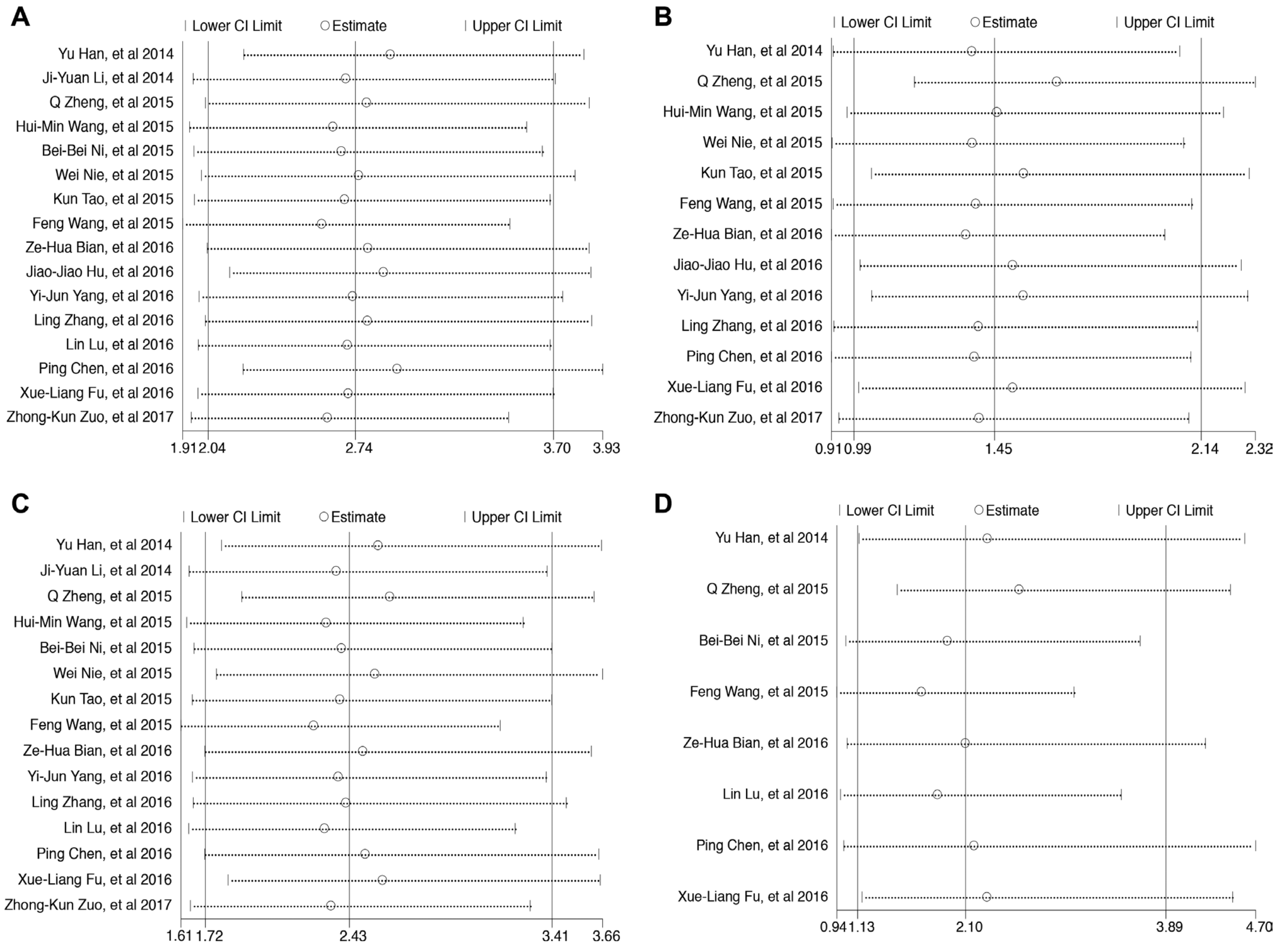

Supplementary Figure 1: Sensitivity analysis of the effect of the individual study on the pooled ORs. (**A**) Sensitivity analysis of clinical stage. (**B**) Sensitivity analysis of tumor size. (**C**) Sensitivity analysis of lymphatic metastasis. (**D**) Sensitivity analysis of distant metastasis.

Supplementary Table 1: The overall characteristics of OS studies in this meta-analysis. See Supplementary_Table_1

Supplementary Table 2: The overall characteristics of clinicopathological studies in this meta-analysis. See Supplementary_Table_2

Supplementary Table 3: The value of quality evaluation estimated on the REMARK guideline. See Supplementary_Table_3

Supplementary Table 4: The Egger's test for potential publication bias in OS analysis

Publication bias	Total	Sample size subgroup		Quality score subgroup		Analysis method subgroup	
		Size ≥ 90	Size < 90	Score ≥ 75	Score < 75	Multivariate analysis	Univariate analysis
No. of studies	19	8	11	8	11	14	5
Egger' test (*p* value)	0.006	0.001	0.186	0.000	0.338	0.000	0.744

Supplementary Table 5: The Egger's test for potential publication bias in clinicopathological analysis

Publication bias	No. of studies	Total Egger' test (*p* value)	Sample size subgroup Egger' test (*p* value)		Quality score subgroup Egger' test (*p* value)	
			Size ≥ 90	Size < 90	Score ≥ 75	Score < 75
Tumor stage	16	0.017	0.054	0.010	0.011	0.429
Tumor size	13	0.622	0.540	0.716	0.869	0.079
Lymphatic metastasis subgroup	15	0.011	0.171	0.002	0.087	0.149
Distant metastasis	8	0.653	0.934	0.377	0.657	0.905
